# The effects of a pre-workout supplement containing caffeine, creatine, and amino acids during three weeks of high-intensity exercise on aerobic and anaerobic performance

**DOI:** 10.1186/1550-2783-7-10

**Published:** 2010-02-15

**Authors:** Abbie E Smith, David H Fukuda, Kristina L Kendall, Jeffrey R Stout

**Affiliations:** 1 Metabolic and Body Composition Laboratory, Department of Health and Exercise Science, University of Oklahoma, Norman, OK 73019, USA

## Abstract

**Background:**

A randomized, single-blinded, placebo-controlled, parallel design study was used to examine the effects of a pre-workout supplement combined with three weeks of high-intensity interval training (HIIT) on aerobic and anaerobic running performance, training volume, and body composition.

**Methods:**

Twenty-four moderately-trained recreational athletes (mean ± SD age = 21.1 ± 1.9 yrs; stature = 172.2 ± 8.7 cm; body mass = 66.2 ± 11.8 kg, VO_2_max = 3.21 ± 0.85 l·min^-1^, percent body fat = 19.0 ± 7.1%) were assigned to either the active supplement (GT, n = 13) or placebo (PL, n = 11) group. The active supplement (Game Time^®^, Corr-Jensen Laboratories Inc., Aurora, CO) was 18 g of powder, 40 kcals, and consisted of a proprietary blend including whey protein, cordyceps sinensis, creatine, citrulline, ginseng, and caffeine. The PL was also 18 g of powder, 40 kcals, and consisted of only maltodextrin, natural and artificial flavors and colors. Thirty minutes prior to all testing and training sessions, participants consumed their respective supplements mixed with 8-10 oz of water. Both groups participated in a three-week HIIT program three days per week, and testing was conducted before and after the training. Cardiovascular fitness (VO_2_max) was assessed using open circuit spirometry (Parvo-Medics TrueOne^® ^2400 Metabolic Measurement System, Sandy, UT) during graded exercise tests on a treadmill (Woodway, Pro Series, Waukesha, WI). Also, four high-speed runs to exhaustion were conducted at 110, 105, 100, and 90% of the treadmill velocity recorded during VO_2_max, and the distances achieved were plotted over the times-to-exhaustion. Linear regression was used to determine the slopes (critical velocity, CV) and y-intercepts (anaerobic running capacity, ARC) of these relationships to assess aerobic and anaerobic performances, respectively. Training volumes were tracked by summing the distances achieved during each training session for each subject. Percent body fat (%BF) and lean body mass (LBM) were assessed with air-displacement plethysmography (BOD POD^®^, Life Measurement, Inc., Concord, CA).

**Results:**

Both GT and PL groups demonstrated a significant (p = 0.028) increase in VO_2_max from pre- to post-training resulting in a 10.3% and 2.9% improvement, respectively. CV increased (p = 0.036) for the GT group by 2.9%, while the PL group did not change (p = 0.256; 1.7% increase). ARC increased for the PL group by 22.9% and for the GT group by 10.6%. Training volume was 11.6% higher for the GT versus PL group (p = 0.041). %BF decreased from 19.3% to 16.1% for the GT group and decreased from 18.0% to 16.8% in the PL group (p = 0.178). LBM increased from 54.2 kg to 55.4 kg (p = 0.035) for the GT group and decreased from 52.9 kg to 52.4 kg in the PL group (p = 0.694).

**Conclusion:**

These results demonstrated improvements in VO_2_max, CV, and LBM when GT is combined with HIIT. Three weeks of HIIT alone also augmented anaerobic running performance, VO_2_max and body composition.

## Background

The study of nutrient timing has become an important and popular aspect of sports nutrition, exercise training, performance, and recovery [1]. The idea of nutrient timing was initiated by post-workout supplementation and has further spread to research on the timing of pre-exercise nutritional strategies [1]. Traditional nutritional interventions prior to training have focused on carbohydrate administration, while more current literature has supported a combination of amino acids, protein, creatine and caffeine as effective supplements for improving performance [2-6]. While the ergogenic effects from these individual ingredients are generally supported, the practical importance of product-specific research has become an area of increasing demand. Paradoxically, product-specific research often tests a blend of ingredients that provides a direct application of the research findings for consumers, but is unable to pinpoint the effects of individual ingredients. Furthermore, integrating nutritional supplements into research designs that use realistic exercise training protocols allows for impactful sport-specific practical applications. Since many team sports, such as football, basketball, hockey and soccer utilize repeated bouts of short sprints separated by active recovery periods, interval running may be applicable to many sports and sensitive to nutritional supplements that are designed to delay high-intensity, exercise-induced fatigue. In fact, evidence exists to support the use of high-intensity interval training (HIIT) strategies to improve performance [7], however, only a few studies have examined HIIT combined with nutritional supplementation [8-13].

The physiological demand of HIIT elicits rapid metabolic and cardiovascular adaptations, including increased exercise performance, muscle buffering capacity, aerobic capacity (VO_2_peak) and fat oxidation [8,14-17]. Furthermore, HIIT results in diminished stores of adenosine tri-phosphate (ATP), phosphocreatine (PCr) and glycogenic substrates as well as the accumulation of metabolites adenosine di-phosphate (ADP), inorganic phosphate (P_i_), and hydrogen ions (H^+^) [18]. Therefore, HIIT may cause several physiological adaptations within a relatively brief training period, making it a practical time-efficient tool to examine training- and supplement-induced changes in performance. Although the work to rest ratio of HIIT protocols vary, the current study and others utilizing a 2:1 work:rest strategy have been effective for improving VO_2_max, time to exhaustion [9,11,19], muscle buffering capacity, and lactate threshold [8]. Additionally, the same HIIT strategy that is used in the present study has been employed to evaluate the effects of creatine [9,10], beta-alanine [11], and sodium bicarbonate [8] supplementation on measures of performance. Therefore, it is possible that the training outcomes measured after a period of HIIT may be sensitive to nutritional supplements that are designed to prolong the acute factors associated with fatigue. More so, the active ingredients in the current pre-workout supplement have potential to improve performance. Caffeine or caffeine containing supplements acting as a central nervous system stimulant [20] have been suggested to augment catecholamine concentrations promoting fat utilization sparing intramuscular glycogen resulting in an improvement in performance [21,22]. PCr, a major component of biological buffering has been reported to be significantly increased with Cr supplementation [23,24]. Increasing total Cr stores can result in greater pre-exercise PCr availability, improved muscle buffer capacity and an acceleration of PCr resynthesis during recovery [25,26]. Additionally, branched chain amino acids (BCAA's; leucine, isoleucine, and valine) are suggested to be the primary amino acids oxidized during intense exercise [27]. When supplementing with BCAAs prior to exercise, research suggests an improvement in protein synthesis, reduction in protein degradation, ultimately improving recovery [27-29].

Interval training is generally used to elicit both anaerobic and aerobic training adaptations due to the large physiological spectrum of demands [30]. The critical power test (CP), originally proposed by Monod and Scherrer [31], characterizes both anaerobic work capacity (AWC) and aerobic parameters (CP). The CP test has been shown to be reliable in measuring aerobic and anaerobic parameters as well as changes with high-intensity training [10,32-34]. Hughson et al. [35] applied the concept of CP to running, which characterized the term critical velocity (CV) as the running-based analogue of CP. Thus, CV is defined as the maximal running velocity that can be maintained for an extended period of time using only aerobic energy stores. In contrast, the anaerobic running capacity (ARC) is the distance that can be run at a maximal velocity using only anaerobic energy sources. As described by Housh et al. [36], the CV test involves a series of runs to exhaustion at various supramaximal running velocities to determine the relationship between time to exhaustion and velocity. The hyperbolic relationship between velocity and time to exhaustion can then be used to calculate total distance (total distance = velocity × time). Plotting total distance as a function of time for each velocity results in a mathematical model to quantify CV (slope of the line) and ARC (y-intercept), which defines the indirect method of evaluating both aerobic and anaerobic capabilities, respectively [35,37].

Recent evidence has shown that interval training with two-minute work bouts, similar to the HIIT in the present study, exerts a significant influence on aerobic abilities (CV), rather than the anaerobic improvements (AWC) demonstrated by the CP test [32,38]. Training at intensities of 100% and 105% of VO_2_peak on a cycle ergometer elicited a 15% [32] and 13% [38] increase in aerobic capacity, respectively. Training at higher intensities for shorter durations (i.e. 60 sec) may be more advantageous for anaerobic improvements [33], although this hypothesis has not been evaluated using the CV test. Likewise, the efficacy of single-ingredient supplements has been assessed using the CP model. For example, creatine supplementation has been shown to improve AWC, which is primarily limited by the amount of energy available from stored ATP and phosphocreatine (PCr) [39]. However, less conclusive evidence is available on the effects of creatine on CP [10,40,41]. It is possible that combining the use of a multi-ingredient, pre-workout supplement with HIIT may further delineate the anaerobic and aerobic demands of training as measured by CV and ARC using the running-based CV test. Therefore, the purpose of the present study was to examine the effects of a pre-workout supplement combined with three weeks of HIIT on aerobic and anaerobic running performance, training volume, and body composition. To date, no one has examined the combined effects of high-intensity interval running with a pre-workout nutritional supplement.

## Methods

### Subjects

Twenty-four moderately-trained men (mean ± SD age = 20.8 ± 2.0 yrs; stature = 175.7 ± 8.3 cm; body mass = 70.9 ± 13.5 kg, VO_2_max = 3.71 ± 0.73 l·min^-1^, percent body fat = 14.0 ± 4.6%) and women (mean ± SD age = 21.5 ± 1.8 yrs; stature = 168.0 ± 7.5 cm; body mass = 60.7 ± 6.5 kg, VO_2_max = 2.57 ± 0.48 l·min^-1^, percent body fat = 24.9 ± 4.4%) volunteered for this study. Table 1 shows the groups-specific demographics. All participants completed a health history questionnaire and signed a written informed consent prior to testing to screen for training habits and prior caffeine and supplement use. All procedures were approved by the University's Institutional Review Board for the protection of human subjects.

**Table 1:** Baseline age (yrs), height (cm), weight (kg) and body fat (%) characteristics.

	Age (yrs)	Height (cm)	Weight (kg)	Body Fat (%)
GT (n = 13)	21.3 ± 0.7	171.7 ± 1.7	66.9 ± 4.1	18.9 ± 2.1

PL (n = 11)	20.8 ± 0.3	172.7 ± 1.8	65.4 ± 2.4	19.1 ± 2.1

	*p = 0.488*	*p = 0.770*	*p = 0.756*	*p = 0.949*

There were no significant differences between groups.

### Research Design

**Figure 1:**
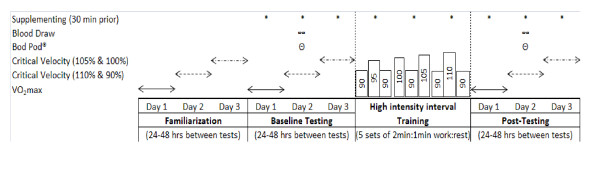
**Study Timeline**.

This study used a randomized, single-blinded, placebo-controlled parallel design. Each subject visited the laboratory on 18 separate occasions, where visits 1-3 were familiarization sessions, visits 4-6 and 16-18 were baseline and post-testing sessions, respectively. All testing sessions were separated by 24-48 hours. Visits 7-15 took place over a three-week period, with three days of training per week. Figure 1 illustrates the timeline for testing and training.

All participants completed a familiarization week of testing, including a maximal graded exercise test (GXT) for the determination of aerobic capacity (VO_2_max) followed by two separate days of runs to exhaustion to determine CV and ARC. These familiarization sessions were implemented to minimize any potential learning effects. After familiarization, participants were randomly assigned to a supplementation group: (a) an active pre-workout supplement (Game Time^®^, GT, n = 13) or (b) placebo (PL, n = 11). The same GXT, CV, and ARC testing that took place during the familiarization sessions were performed at baseline (pre-training) and post-training (Figure 1).

All participants were instructed to maintain their current dietary habits throughout the duration of the study. Furthermore, participants were asked to refrain from caffeine and any vigorous activity for 24 hours prior to any testing session.

### Body Composition Assessments

Air displacement plethysmography (ADP; BOD POD^®^, Life was Measurement, Inc., Concord, CA) was used to estimate body volume after an eight-hour fast at baseline and post-testing. Prior to each test, the BOD POD was calibrated according to the manufacturer's instructions with the chamber empty and using a cylinder of known volume (49.55 L). The participant, wearing only Spandex shorts or tight-fitting bathing suit and swimming cap, entered and sat in the fiberglass chamber. The BOD POD was sealed and the participant breathed normally for 20 seconds while BV was measured. The subjects' weight and body volume were measured and used to determine percent body fat (%BF), fat mass (FM, kg), and lean body mass (LBM, kg) using the revised formula of Brozek et al.[42]. Previous test-retest reliability data for ADP from our laboratory indicated that, for 14 young adults (24 ± 3 yrs) measured on separate days, the ICC was 0.99 with a SEM of 0.47% fat.

### Supplementation

The caloric values and nutrient compositions of the GT and PL supplements are listed in Table 2. On each of the testing and training days the participants ingested the GT or PL in the laboratory 30 minutes prior to testing on an empty stomach (subjects were instructed not to eat within 4 hours prior to their laboratory visits). Since the GT and PL supplements were in powder form, the investigators mixed the contents of the GT or PL packets with 8-12 oz of cold tap water in a white cup prior to the participant's arrival. After the mixture was consumed, a stopwatch was used to precisely allow 30 minutes after consumption prior to the initiation of the testing or training. The participants did not consume the GT or PL drinks on the rest days; therefore, supplementation only occurred prior to the in-laboratory testing or training visits.

**Table 2:** Pre-workout supplement ingredients for the active (GT) and placebo (PL) groups.

GT Supplement	PL Supplement
Calories: 40	Calories: 40
Calories from Fat: 5	Calories from Fat: 0

Total Fat: 0 g	Maltodextrin: 17 g
Cholesterol: 20 mg	Proprietary Blend: 3 g
Sodium: 270 mg Total Carbohydrates: 2 g Sugars: 2 g	Natural and artificial flavors, citric acid, sucralose, acesulame potassium, Red#40
Protein: 8 g	
Vitamin A: 0%	
Vitamin C: 0%	
Calcium: 4%	
Vitamin B12: 2000%	
Vitamin B6: 500%	
Iron: 0%	

Proprietary Blend: 2100 miligrams Cordyceps sinensis, Arginine AKG, Kre-Alkalyn, Citrulline AKG, Eleutherococcus senticosus, Taurine, Leucine, Rhodiola Rosea, Sodium Chloride, Valine, Isoleucine, Caffeine, Whey Protein Concentrate	

### Determination of VO_2_max

All participants performed a GXT to volitional exhaustion on a treadmill (Woodway, Pro Series, Waukesha, WI) to determine VO_2_max. Based on the protocol of Peake et al.[43], the initial GXT velocity was set at 10 km/h at a 0% grade and increased 2 km·h^-1 ^every two minutes up to 16 km·h^-1^, followed by 1 km·h^-1^increments per minute up to 18 km·h^-1^. The gradient was then increased by 2% each minute until VO_2_max was achieved. Open-circuit spirometry was used to estimate VO_2_max (l·min^-1^) with a metabolic cart (True One 2400^® ^Metabolic Measurement System, Parvo-Medics Inc., Sandy, UT) by sampling and analyzing the breath-by-breath expired gases. The metabolic cart software calculated VO_2 _and determined the VO_2_max value for each GXT.

### Critical Velocity

To determine CV and ARC, the linear Total Distance (TD) model described and evaluated by Florence and Weir et al [44] was used:

Where the total distance achieved during each run to exhaustion (TD; y axis) was plotted over the time-to-exhaustion (t; x axis), and linear regression was used to calculate the y-intercept (ARC) and the slope (CV).

Four treadmill runs to exhaustion were performed to establish the distance-time relationships for the TD model for each subject. Each participant ran at 90%, 100%, 105%, and 110% of the treadmill velocity (km·h^-1^) that corresponded with their VO_2_max score. The time-to-exhaustion (s) and distance achieved (km) was recorded for each run.

### High-intensity interval training

After baseline testing, participants completed three weeks of high-intensity interval training (HIIT) for three days per week using a fractal periodization scheme to adjust the training velocities. Each training session consisted of five sets of two-minute running bouts with one minute of rest between each bout. The total running duration (s) and velocity (km·h^-1^) during each training session was recorded and used to calculate total training volume (km). Training was performed on the same treadmill used for the GXTs (Woodway, Pro Series, Waukesha, WI). Figure 1 shows the relative treadmill velocities used during the training period. The training intensity began at 90% of the velocity achieved during the baseline VO_2_max test and progressed in an undulating manner, reaching a maximum of 110% by the end of the three-week training period.

### Statistical Analyses

Five separate two-way, mixed factorial ANOVA models (2 × 2; time [pre- vs. post-training] × group [GT vs. PL]) were used to analyze the raw CV, ARC, VO_2_max, %BF, FM, and LBM data. For significant interactions, independent- or dependent-samples t-tests were used as post-hoc tests. For training volume, the sum of training distances for all nine training visits was calculated for each subject, and an independent-sample t-test was used to examine the means of the total training volume values (km). In addition, independent-sample t-tests were used to determine group mean differences (GT vs. PL) during the pre-training testing sessions.

**Figure 2:**
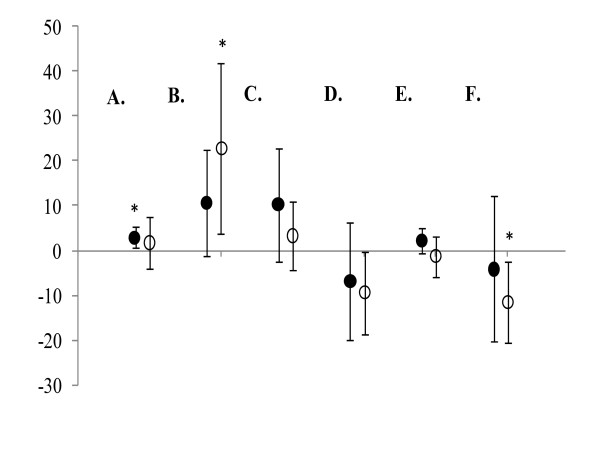
**Mean percent change scores ± 95% confidence intervals for (A) critical velocity, (B) anaerobic running capacity, (C) aerobic capacity, (D) percent body fat, (E) lean body mass, and (F) fat mass**. Black circles = GT group; White circles = PL group. * indicates a significant difference when 0 is outside of the 95% confidence interval.

**Figure 3:**
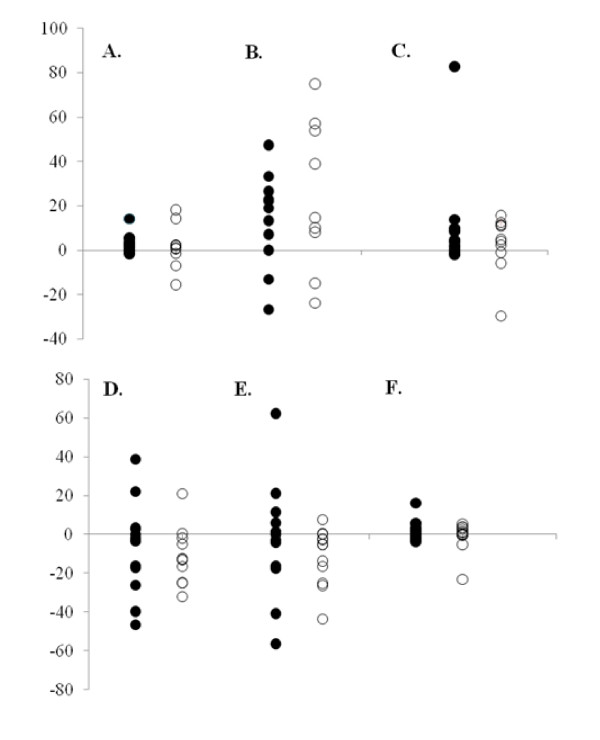
**Percent change scores from pre- to post-training for each individual participant for (A) critical velocity, (B) anaerobic running capacity, (C) aerobic capacity, (D) percent body fat, (E) fat mass and (F) lean body mass**. Black circles = GT group; White circles = PL group.

Except for training volume, percent change scores were calculated for each participant from pre- to post-training for CV, ARC, VO_2_max, %BF, FM, and LBM. These percent changes scores were averaged separately for the GT and PL groups and 95% confidence intervals were constructed around the mean percent change scores (Figure 2). When the 95% confidence interval includes zero, the mean percent change score is no different from zero, which can be interpreted as no statistical change (p > 0.05). However, if the 95% confidence interval does not include zero, the mean percent change for that variable can be considered statistically significant (p ≤ 0.05). In addition, individual response graphs were created and plotted to illustrate how each subject responded from pre- to post-training (Figure 3).

A type I error rate that was less than or equal to 5% was considered statistically significant for all analyses. ANOVA models and t-tests were computed using SPSS (Version 14.0, SPSS Inc., Chicago, Ill), and the 95% confidence intervals and individual response graphs were calculated and created in Microsoft Excel (Version 2007, Microsoft Corporation; The Microsoft Network, LLC, Richmond, WA).

## Results

Table 3 contains the means and standard errors for each of the dependent variables (CV, ARC, VO_2_max, %BF, FM, and LBM). In addition, there were no significant differences (p > 0.05) between the GT and PL groups at the pre-training testing session.

**Table 3:** Mean ± SE values from pre- to post-training for critical velocity (CV), anaerobic running capacity (ARC), maximal oxygen consumption (VO_2_max), percent body fat (%BF), fat mass (FM) and lean body mass (LBM) for GT and PL.

	CV (km/hr)		ARC (km)		VO_2_max (l·min^-1^)		VO_2_max (ml·kg·min)	
	**Pre**	**Post**	**Pre**	**Post**	**Pre**	**Post**	**Pre**	**Post**

GT (n = 13)	12.4 ± 0.8	12.8 ± 0.8	0.2 ± 0.01	0.2 ± 0.02	3.1 ± 0.3	3.65 ± 0.2*	47.9 ± 3.4	56.2 ± 2.7*
PL (n = 11)	10.7 ± 0.5	10.9 ± 0.6	0.2 ± 0.03	0.3 ± 0.04	3.1 ± 0.2	3.2 ± 0.3*	56.5 ± 2.1	45.3 ± 2.3*

	**%BF**		**FM (kg)**		**LBM (kg)**			

	**Pre**	**Post**	**Pre**	**Post**	**Pre**	**Post**		

GT (n = 13)	18.9 ± 2.5	17.7 ± 2.1	12.7 ± 1.9	12.0 ± 1.7	54.2 ± 3.5	55.4 ± 3.7		
PL (n = 11)	19.1 ± 1.8	17.1 ± 1.9	12.4 ± 1.1	10.6 ± 1.1	53 ± 2.7	52.4 ± 3.2		

*indicates a significant difference over time (p < 0.05).

### ANOVA Models

For CV, there was no time × group interaction (p = 0.256) and no main effect for time (p = 0.507), but there was a main effect for group (p = 0.036). CV for the GT group was greater than the PL group at the pre- and post-training testing sessions.

For ARC, there was no time × group interaction (p = 0.183) and no main effects for time (p = 0.093) or group (p = 0.053).

For VO_2_max, there was no time × group interaction (p = 0.391) and no main effect for group (p = 0.258), but there was a main effect for time (p = 0.028). VO_2_max increased from pre- to post-training for the GT and PL groups.

For %BF, there was no time × group interaction (p = 0.481) and no main effects for time (p = 0.178) or group (p = 0.864).

For FM, there was no time × group interaction (p = 0.335) and no main effects for time (p = 0.305) or group (p = 0.583).

For LBM, there was no time × group interaction (p = 0.386) and no main effects for time (p = 0.694) or group (p = 0.615).

**Figure 4:**
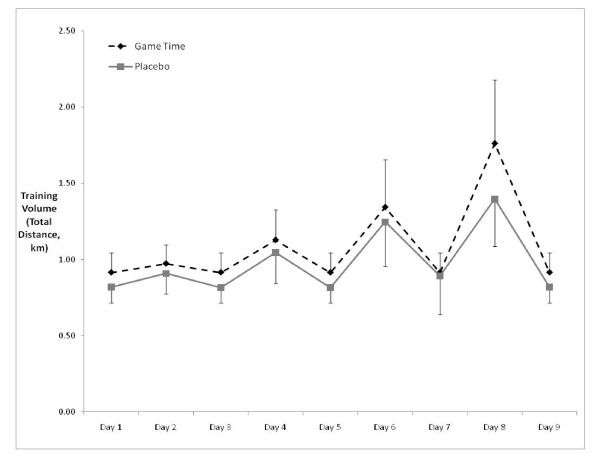
**Training volume for the GT and PL groups across the nine day training session**.

Total training volume for the GT group was greater than (p = 0.041) the PL group. (Figure 4).

### 95% Confidence Intervals

CV increased from pre- to post-training for the GT group (2.9% increase), but did not change for the PL group (1.7% increase) (Figure 2-A). However, Figure 2-B shows that ARC did not change from pre- to post-training for the GT group (10.6% increase), but did increase for the PL group (22.9% increase). VO_2_max did not change from pre- to post-training for either the GT (10.3% increase) or PL (3.3% increase) groups (Figure 2-C). For body composition, %BF did not change for either the GT (6.7% decrease) or PL (9.4% decrease) groups (Figure 2-D), LBM did not change for either the GT (2.8% increase) or PL (1.3% decrease) groups (Figure 2-E), and FM did not change for either the GT (4.1% decrease) or PL (11.6% decrease) groups (Figure 2-F) from pre- to post-training.

### Individual Responses

For CV, 10 out of 13 (77%) subjects increased in the GT group, whereas only 7 of 11 (64%) increased in the PL group (Figure 3A). Eight subjects increased in the GT (62%) and PL (73%) groups for ARC (Figure 3B). For VO_2_max, 10 increased in the GT group (77%), and 8 increased in the PL group (73%) (Figure 3C).

Nine subjects in the GT group (69%) and 8 subjects in the PL group (73%) decreased in %BF from pre- to post-training (Figure 3D). Similarly, 8 subjects in both groups (62% for GT and 73% for PL) showed a decrease in FM (Figure 3E). LBM increased for 9 subjects in the GT group (69%), while only 6 subjects increased in the PL group (55%) (Figure 3F).

## Discussion

The results of the present study indicated that acute ingestion of the current pre-exercise drink (GT) containing a combination of cordyceps sinensis, caffeine, creatine (Kre-Alkalyn^®^), whey protein, branched chain amino acids, arginine AKG, citrulline AKG, rhodiola, and vitamin B6 and B12 may improve running performance over a 3-week training period. When combined with HIIT, GT ingestion improved CV, VO_2_max, lean body mass, and total training volume when compared to the PL and HIIT group. In addition, although not significant, the fact that LBM changes were positive for the GT group and negative for the PL group (Figure 2-E) suggests that GT may aid in maintaining LBM during the course of HIIT for three weeks.

While this may be the first study to examine a pre-workout supplement in combination with HIIT, previous research has examined the efficacy of similar, separate ingredients on exercise training and performance. However, since most previous studies examine blended supplements that often include various ingredients and dose combinations, it is difficult to directly compare many previous studies. One primary ingredient in the GT supplement, caffeine, has been used as an effective ergogenic aid by acting as a stimulant, reducing feelings of fatigue, and increasing times to exhaustion [22,45-47]. Caffeine has been shown to primarily influence longer-duration endurance exercise by 20-50% [48] and resting metabolic rate [45,49-51]. The benefits of caffeine supplementation for higher-intensity exercise, similar to those in the current study (90%-115% VO_2_max), are less conclusive [52,53]. For example, assessing anaerobic power using a Wingate test after a range of caffeine doses (3.2-7 mg/lb) resulted in no improvements [52,53] while Anselme et al. demonstrated a 7% increase in anaerobic power after 6 mg/kg of caffeine consumption [54]. In addition, a recent report by Wiles et al. demonstrated improvements in performance during a bout of short-duration, high-intensity cycling and mean power output following 5 mg/kg of caffeine [55]. The results of the present study indicated that the pre-exercise GT drink improved aerobic performance (CV) and training volume, but did not alter the ARC. It is possible that the caffeine in GT may be partly responsible for the increases in CV and training volume. However, the independent effects of caffeine cannot be directly assessed in the present study.

Previous studies have suggested that the ergogenic effects of caffeine may be proportional to the amount of caffeine administered [56-58]. Most studies have utilized 3-9 mg/kg of caffeine when demonstrating improvements in performance [48], while one study showed that as little 2 mg/kg increased cycling performance [58]. Yet another study demonstrated that 201 mg of caffeine was not sufficient for increasing run time to exhaustion [59]. In the present study, the pre-exercise GT supplement contained only 100 mg of caffeine in one serving. Since the range of body mass values for the participants in the present study was 46.1 kg to 108.9 kg, the relative caffeine doses were 1.0 - 2.2 mg/kg, which is lower than the previously suggested ergogenic doses. Therefore, although caffeine may have contributed to improvements in aerobic performance and training volume in the present study, it is possible that there were synergistic effects from other GT ingredients.

One concern about the ergogenic doses of caffeine is that relatively high levels of urinary caffeine concentrations are banned by both the National Collegiate Athletics Association (NCAA) and the International Olympic Committee (IOC). The NCAA and IOC limits for urinary caffeine concentrations are 15 μg/ml and 12 μg/ml, respectively. In a well-controlled study [60] the average urinary concentration of caffeine was 14 μg/ml after the ingestion of 9 mg/kg. In an earlier study, Pasman et al. (1995) demonstrated that 9 and 13 mg/kg of caffeine consumption resulted in urinary caffeine concentrations that exceeded the International Olympic Committee's (IOC's) limit of 12 μg/ml in some subjects. However, 5 mg/kg of caffeine did not exceed or even approach 12 μg/ml in any subject [61]. Since the relative caffeine dose range for the GT supplement in the present study was 1.0 - 2.2 mg/kg for an absolute dose of 100 mg of caffeine per serving, it is highly unlikely that the caffeine in GT would cause urinary concentrations anywhere near the limits set forth by the NCAA or IOC. Therefore, although not tested specifically in this study, the GT supplement may be safe for consumption by NCAA and IOC athletes as it pertains to caffeine concentrations.

A large amount of literature exists demonstrating that short-term high-dose (20 g/day for 5-7 days) creatine supplementation is effective for increasing total muscle phosphocreatine stores [23,24] and improving maximal intermittent exercise [23,25,62-64] and lean body mass [64-68]. However, the data on short-term low-dose creatine supplementation is less supported, with a minimum of 3 g/day for at least 28 days necessary to elicit increases in muscle creatine stores [69]. The current pre-workout GT drink provided 1.5 g/day of creatine on testing and training days only for a total of 15 days, which was below the minimum recommended dose. A similar study by Thompson and colleagues used a comparable combination of training (swimming) and 2 g of creatine daily for six weeks and demonstrated no effects of the creatine supplementation or training on muscle creatine concentration, anaerobic performance, or aerobic indices [70]. Thus, although the creatine content of the GT supplement may not fully explain the improvements in CV and training volume, the combination with the other GT ingredients may have been influential for intermittent recovery between sprint bouts as well as helping to maintain LBM.

The BCAAs in GT may have also played a role in improving CV and training volume as well as maintaining LBM. BCAAs may be the primary amino acids oxidized during intense exercise [27] and have been suggested as fundamental for protein synthesis [27-29]. Studies have demonstrated that the ingestion of BCAA supplements prior to exercise has augmented protein synthesis and reduced protein degradation, which may ultimately enhance recovery time [27,29]. Furthermore, BCAAs may conceivably enhance performance in all-out running, similar to the current study by improving mental focus allowing participants to run harder and longer [71,72]. Again, however, the GT supplement contained approximately 1 g of BCAAs which is lower than other effective dosing protocols (7.5-12 g). There was also approximately 9 g of whey protein concentrate in the GT supplement. Although whey protein has not been directly shown to improve running performance when consumed a priori, the fact that whey protein also contains relatively high concentrations of the BCAAs may indirectly suggest that the BCAAs in combination with whey protein may influence performance by enhancing recovery between training bouts and maintaining LBM [73-76].

Cordyceps sinensis (or simply *cordyceps*) is commonly used in traditional Chinese medicine, and it is derived from a fungus that grows on several species of caterpillars at relatively high altitudes[77]. It has been suggested that cordyceps may be an anti-oxidant during intense exercise [78] and may also improve VO_2_max [79]. In two reviews by Zhu et al. [77,80], it was suggested that cordyceps sinensis may act through the autonomic nervous system to improve respiration, blood flow, and tissue oxygenation. One study has demonstrated improvements in VO_2_max in sedentary men [79] with relatively high doses (4.5 g/d for 6 weeks) of cordyceps. However, with lower doses (2.5 g) similar to what is found in GT in the present study, there were no ergogenic effects of cordyceps reported in previous studies on VO_2_max [81-83] in healthy, active men. Thus, given the conflicting evidence, cordyceps may be another ingredient in GT that acted synergistically to improve CV and training volume in the present study.

The role that the remaining ingredients in the GT supplement (ex. Citrulline and rhodiola) may play is not completely evident. Citrulline is a non-essential amino acid that may increase lactate absorption, enhance ATP resynthesis, and delay fatigue during intense exercise [84,85]. While evidence is limited in humans, citrulline may have influenced ATP/PCr resynthesis during HIIT bouts thereby enhancing the training volume. Furthermore, rhodiola may act as a stimulant to optimize serotonin and dopamine levels [86]. Acute supplementation (i.e., 2 days) has been shown to increase time to exhaustion and VO_2_peak by acting as an antioxidant and reducing the perception of fatigue [87-90]. Together these ingredients may have positively influenced CV and training volume, however, this speculation cannot be proven in the current study.

## Conclusion

In conclusion, the results of this study indicate that the acute ingestion of the pre-exercise GT supplement containing 100 mg of caffeine, 1.5 g creatine, 1 g BCAAs, 9 g whey protein, 2.5 g of cordyceps sinensis and a combined 0.75 g of citrulline and rhodiola, taken prior to HIIT for three weeks can significantly improve CV and total training volume when compared to HIIT and PL. Furthermore, the maintenance of and trend toward an improvement in LBM suggests that GT may be helpful in maintaining lean mass during intense training periods. Although there was not a single ingredient in GT that could solely account for the improvements, it is likely that the combination of relatively low doses of several ingredients (caffeine, creatine, BCAAs, whey protein, and cordyceps) may be responsible for the increases in aerobic performance, training volume, and the maintenance of lean mass.

## Competing interests

The authors declare that they have no competing interests.

## Authors' contributions

AES was the primary author of the manuscript and played an important role in study design, data collection and assessment. DHF and KLK played an important role in data collection and manuscript preparation. JRS was the senior author and played an important role in the grant procurement, study design, data analysis and manuscript preparation. All authors have read and approved the final manuscript.
